# Case Report: Drug-coated balloon after intravascular lithotripsy for the treatment of severely calcified *de novo* coronary artery lesion

**DOI:** 10.3389/fcvm.2024.1470785

**Published:** 2024-11-18

**Authors:** Toru Misawa, Tetsumin Lee, Takashi Ashikaga, Toshihiro Nozato, Taishi Yonetsu, Tetsuo Sasano

**Affiliations:** ^1^ Department of Cardiology, Japanese Red Cross Musashino Hospital, Tokyo, Japan; ^2^ Department of Cardiovascular Medicine, Tokyo Medical and Dental University, Tokyo, Japan

**Keywords:** severely calcified lesions, intravascular lithotripsy, drug-coated balloon, percutaneous coronary intervention, optical coherence tomography

## Abstract

In patients undergoing percutaneous coronary intervention (PCI), severely calcified lesions remain a great challenge even in the drug-eluting stent (DES) era. Intravascular lithotripsy (IVL) is effective for modification of severely calcified lesions prior to DES implantation. However, the efficacy of PCI with drug-coated balloon (DCB) following IVL has not been fully elucidated. Here, we present a case of severely calcified *de novo* coronary artery lesion successfully underwent PCI with DCB following IVL under optical coherence tomography (OCT) guidance as well as mid-term follow-up OCT. DCB following IVL might be a potential revascularization strategy for patients with heavily calcified *de novo* coronary artery lesions.

## Introduction

Severely calcified lesions remain a great challenge for percutaneous coronary intervention (PCI) even in the drug-eluting stent (DES) era, because coronary calcification interferes with the delivery and expansion of the stent (1). Debulking devices such as rotational atherectomy (RA) or orbital atherectomy (OA) have been used to facilitate the lesion preparation of heavily calcified plaques, leading to larger stent area. Previous studies demonstrated that the efficacy and the safety of drug-coated balloon (DCB) treatment after RA for calcified coronary lesions might be comparable to those of DES following RA (2, 3). Furthermore, clinical outcome of OA combined with DCB for coronary calcification has been reported to be comparable to those of OA followed by DES (4).

Recently, the effectiveness and the safety of intravascular lithotripsy (IVL) prior to DES implantation have been reported and IVL devices have been introduced to clinical practice to obtain adequate modification of severe calcification (5–7). Moreover, IVL indications are quickly increasing over the last years not only for the treatment of coronary stenosis but even for peripheral intervention, for the treatment of severe aortic stenosis or severely calcified carotid artery stenosis (8–10). However, it remains unclear whether DCB following IVL treatment can be used as an alternative strategy for the revascularization of heavily calcified coronary artery lesions. Herein, we presented a patient with a severely calcified *de novo* coronary lesion that successfully underwent PCI with DCB following IVL under optical coherence tomography (OCT) guidance as well as mid-term follow-up OCT.

## Case presentation

**Figure 1:**
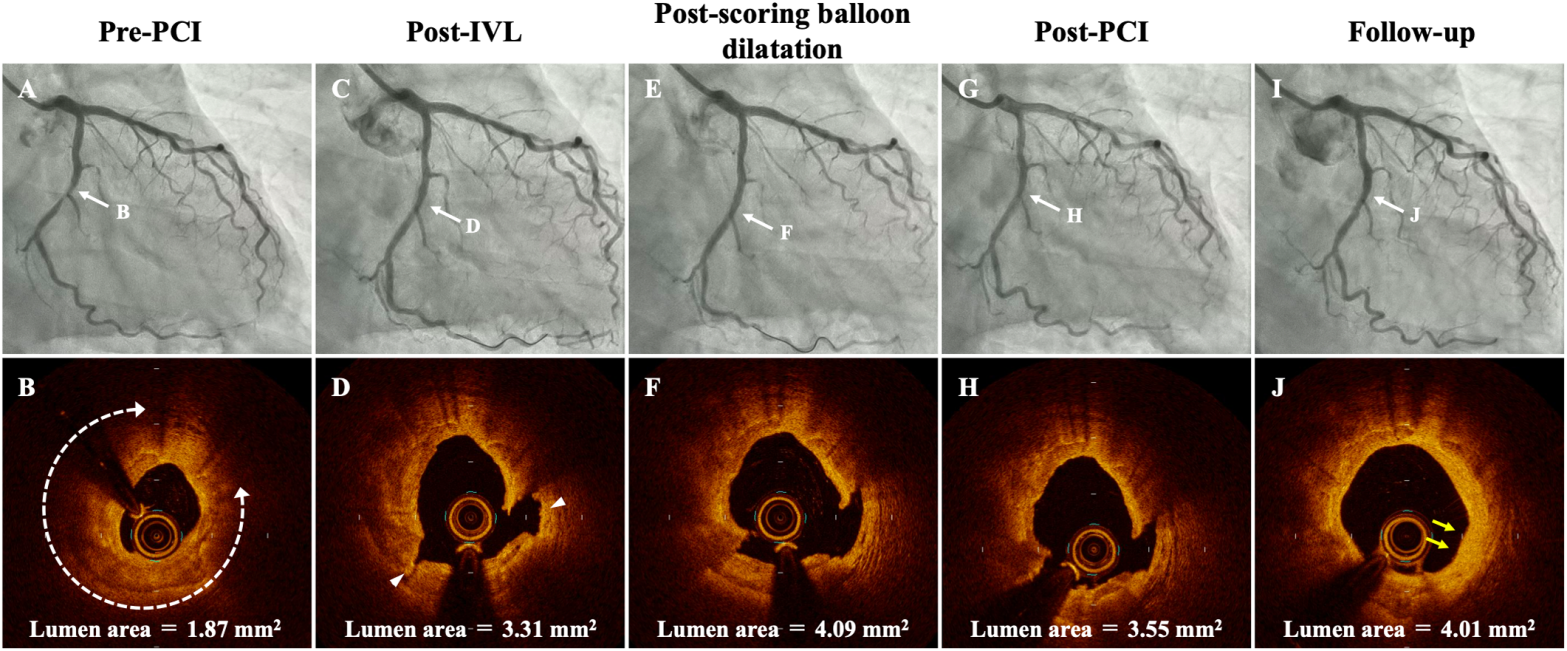
Serial coronary angiogram (CAG) and corresponding optical coherence tomography (OCT) images of the patient. **(A,B)** Pre-PCI CAG and OCT shows a severely calcified coronary stenosis in the mid-left circumflex artery (LCX) with 279° of calcium angle (white-dotted double headed arrows), 0.81 mm of calcium thickness, 5.1 mm of calcified segment length, and 1.87 mm^2^ of minimum lumen area. **(C,D)** Post-IVL CAG and OCT demonstrates good expansion at the lesion with two calcium fractures (arrowhead). **(E,F)** Post-scoring balloon dilatation CAG and OCT showed that lumen area was significantly enlarged. **(G,H)** CAG and OCT after PCI shows no significant residual stenosis and acceptable lumen area. **(I,J)** Follow-up CAG and OCT at 8 months after the procedure. Restenosis is not observed at the mid-LCX, and OCT demonstrates homogeneous neointima with late lumen enlargement in the modified calcified lesions (yellow arrows). IVL, intravascular lithotripsy; PCI, percutaneous coronary intervention.

A 63-year-old male with past medical history of diabetes mellitus and dyslipidemia, was admitted to our hospital for non-ST-elevation myocardial infarction with subacute occlusion of the distal right coronary artery (RCA) and severely calcified coronary stenosis in the mid-left circumflex artery (LCX) (Figure 1A). A few days after primary PCI of RCA, we performed staged PCI of LCX. The left coronary artery was cannulated by a 6F JL3.5 guide catheter via the left distal radial artery approach. A 0.014-inch guidewire was advanced into the distal LCX. Pre-PCI OCT showed a severely calcified coronary stenosis in the mid-LCX with 279° of calcium angle, 0.81 mm of calcium thickness, 5.1 mm of calcified segment length, and 1.87 mm^2^ of minimum lumen area (Figure 1B). Because of the OCT-based calcium score of 4 with 2.8 mm of proximal reference lumen diameter and 2.3 mm of distal reference lumen diameter (11), we decided to use a 2.5 mm × 12 mm IVL balloon (Shockwave C2, Shockwave Medical, Inc. Santa Clara, CA, USA) to modify the heavily calcified plaque. The IVL balloon was deployed to the calcified lesion and inflated with 4 atm, then 10 pulses were delivered at a rate of 1 pulse per second, after which the balloon was inflated further to 6 atm. We stopped IVL at 60 pulses because severely calcified coronary lesion was fully enlarged by 60 pulses of IVL on coronary angiogram (CAG). Post-IVL CAG and OCT demonstrated the improvement of the stenotic lesion with the multiple fracture in almost circumferential thick calcified plaques (Figures 1C,D). According to the proximal reference lumen diameter as 2.8 mm on OCT imaging, a 2.75 mm × 13 mm scoring balloon dilatation was then performed for further lumen enlargement (Figures 1E,F). After scoring balloon dilatation, acceptable angiographic and OCT results, which include thrombolysis in myocardial infarction grade 3 flow, residual stenosis ≤30%, and the absence of major coronary dissection without hematoma were observed. We considered the optimal lesion preparation was obtained as reported in the expert consensus statement (12). Thus, a 2.5 mm × 30 mm paclitaxel-coated balloon (SeQuent Please NEO; B. Braun, Melsungen, Germany) was used as a final device, because of the distal reference lumen diameter as 2.3 mm on OCT imaging. Post-PCI CAG and OCT findings demonstrated acceptable lumen expansion with thrombolysis in myocardial infarction grade 3 coronary flow (Figures 1G,H). At 8 months later follow-up CAG, there was no restenosis in the LCX (Figure 1I). In addition, follow-up OCT showed that calcified plaque with fracture was replaced by homogeneous neointima with late lumen enlargement (Figure 1J).

## Discussion

We reported a case of heavily calcified stenosis treated with IVL followed by DCB. To the best of our knowledge, this is the first case report demonstrating the efficacy of stent-less strategy with DCB following IVL by mid-term OCT assessment.

Severely calcified lesions remain a significant challenge in patients undergoing PCI. Although the optimal strategy for severely calcified lesions was controversial, recent studies reported that clinical outcome after PCI using debulking devices prior to DCB treatment for severe calcification was not inferior compared with DES implantation (2–4). Additionally, late lumen loss observed after DES implantation was significantly larger than that observed after DCB treatment in patients with severe calcification (2, 4). While RA or OA can reduce the calcium burden resulted in the luminal area gain, these effect for calcific plaque modification is limited by guidewire bias (13), and the peri-procedural complications remain a great challenge (14).

IVL is a novel therapy for the treatment of severely calcified plaque by delivering circumferential pulsatile acoustic pressure waves to modify calcification (5). Instead of not ablating and reducing calcified plaque, lesion modification using IVL is not dependent on guidewire bias and no extensive training for IVL therapy is required. Previous clinical studies revealed the efficacy and safety of IVL prior to DES implantation in severely calcified lesions (5–7). Additionally, IVL is safe and effective to achieve lumen gain and stent dimensions in underexpanded stents due to heavily calcified lesions (15). Although the use of IVL prior to DCB for the treatment of severely calcified lesions remains off-label, acute luminal gain with calcium fracture by IVL theoretically may positively affect clinical outcome after stent-less PCI using IVL. The International DCB Consensus Group showed that the DCB-only strategy was feasible in case with acceptable angiographic result after optimal lesion preparation (16).

In the present case, fractured calcification was replaced by homogeneous neointima with late lumen enlargement, which was in line with the previous case report, showing that calcified plaque with fracture was replaced by homogeneous neointima 6 months after PCI using DCB following OA (17). It is possible that calcium fracture created by IVL may lead to increase drug penetration and then facilitate morphological changes in severely calcified plaque, resulting in acceptable luminal gain in the late phase. Therefore, DCB following IVL strategy might be clinically applicable if IVL could create the calcium fracture and thus achieve adequate calcium modification and acceptable angiographic result.

## Conclusions

Stent-less PCI by using a combined IVL and DCB therapy might be a potential revascularization strategy for patients with heavily calcified *de novo* coronary artery lesions of small vessels. Further studies are needed to definitively address the efficacy of this strategy.

## Data Availability

The raw data supporting the conclusions of this article will be made available by the authors, without undue reservation.
